# Neuropsychiatric Symptoms and Hearing Loss in Dementia: Unmet Need and Opportunity for Intervention

**DOI:** 10.1093/geroni/igab046.324

**Published:** 2021-12-17

**Authors:** Carrie Nieman, Alexander Kim, Emmanuel Garcia Morales, Constantine Lyketsos, Nicholas Reed, Valerie Cotter, Sara Mamo, Esther Oh

**Affiliations:** 1 Johns Hopkins University School of Medicine, Baltimore, Maryland, United States; 2 Johns Hopkins Bloomberg School of Public Health, Baltimore, Maryland, United States; 3 Johns Hopkins Bayview, School of Medicine, Baltimore, Maryland, United States; 4 Johns Hopkins School of Nursing and School of Medicine, Baltimore, Maryland, United States; 5 University of Massachusetts Amherst, Amherst, Massachusetts, United States

## Abstract

Hearing loss is one of the most common comorbidities among persons with dementia, with prevalence of 60->90%. Most go untreated and disparities exist. However, sensory impairment may influence the health of individuals and care partners. We will share findings from a clinic-based cohort of persons with dementia (n=101). Controlling for demographic and clinical factors, we found that every 10 decibel increase in hearing loss was associated with nearly an additional neuropsychiatric symptom (b = 0.7 per 10 dB; p = 0.01) and 1.3-point increase in severity (b = 1.3 per 10 dB; p = 0.04). These findings provide the first estimates that utilize objective audiometry. Furthermore, hearing aid use appeared to be protective. Hearing care may represent an additional, but underutilized, non-pharmacological intervention. We will discuss these findings in the context of the epidemiology of hearing loss in dementia and highlight new opportunities for managing hearing loss through community-based approaches.

